# Static and Dynamic Analysis of Electrostatically Actuated MEMS Shallow Arches for Various Air-Gap Configurations

**DOI:** 10.3390/mi12080930

**Published:** 2021-08-05

**Authors:** Hassen M. Ouakad, Nouha Alcheikh, Mohammad I. Younis

**Affiliations:** 1 Mechanical and Industrial Engineering Department, College of Engineering, Sultan Qaboos University, P.O. Box 33, Al-Khoudh, Muscat 123, Oman; 2 Physical Science and Engineering Division, King Abdullah University of Science and Technology, Thuwal, Jeddah 23955-6900, Saudi Arabia; nouha.alcheikh@kaust.edu.sa

**Keywords:** MEMS, shallow arch, resonators, response, sensitivity, tunability

## Abstract

In this research, we investigate the structural behavior, including the snap-through and pull-in instabilities, of in-plane microelectromechanical COSINE-shaped and electrically actuated clamped-clamped micro-beams resonators. The work examines various electrostatic actuation patterns including uniform and non-uniform parallel-plates airgap arrangements, which offer options to actuate the arches in the opposite and same direction of their curvature. The nonlinear equation of motion of a shallow arch is discretized into a reduced-order model based on the Galerkin’s expansion method, which is then numerically solved. Static responses are examined for various DC electrostatic loads starting from small values to large values near pull-in and snap-through instability ranges, if any. The eigenvalue problem of the micro-beam is solved revealing the variations of the first four natural frequencies as varying the DC load. Various simulations are carried out for several case studies of shallow arches of various geometrical parameters and airgap arrangements, which demonstrate rich and diverse static and dynamic behaviors. Results show few cases with multi-states and hysteresis behaviors where some with only the pull-in instability and others with both snap-through buckling and pull-in instabilities. It is found that the micro-arches behaviors are very sensitive to the electrode’s configuration. The studied configurations reveal different possibilities to control the pull-in and snap-through instabilities, which can be used for improving arches static stroke range as actuators and for realizing wide-range tunable micro-resonators.

## 1. Introduction

Microelectromechanical based systems (MEMS) have attracted considerable attention in the engineering and science community due to their numerous advantages, such as miniature designs with high sensitivity, resolution and quality factor, and more importantly low power consumption [1,2,3,4]. Among the attractive MEMS designs are those based on initially curved micro-beams (Arc-shaped, COSINE-shaped, V-shaped, etc.). These designs have been extensively employed in many applications, such as smart sensors, actuators, switches, filters, and energy harvesters. Among the various actuation mechanisms, the parallel-plate electrostatic configuration represents the most effective one for its simplicity, low power consumption, compactness, high operating speed, quick switching time, and the compatibility with standard electronics fabrication processes [5,6].

An electrostatically actuated curved micro-beam design comprises an initially curved flexible electrode (the arch) and a stationary one separated by an airgap in a parallel-plate arrangement [7]. When a DC voltage is applied between the electrodes, it generates an attractive force, and therefore the movable electrode starts to move toward the fixed one. Increasing the DC load further, the attractive force increases and therefore the movable plate moves even closer to the stationary electrode. When the DC amplitude surpasses a threshold value where the attractive force is greater than the movable micro-beam elastic restoring force, an instability occurs making the deformable electrode to either snap (flip its curvature to the opposite direction) or to pull-in and collapse on the fixed electrode (short-circuit) [8].

Electrically actuated and curved micro-beams can experience either or both the snap-through and pull-in instabilities. Therefore, it is important to predict the critical values leading to these instabilities for better and robust designs, where in some these are to be avoided and are essential in others. Indeed, in micro-switching applications, the pull-in behavior is desirable and essential for greater switching performances [9,10]. In contrary, this instability is to be avoided in applications involving resonating and sensing operations [11,12]. The co-existence of states under the same actuating force showlots of potential in applications ranging from sensors, filters, harvesters, and large-stroke switches [13,14,15,16,17,18,19]. The motion between the two stable states is known as the snap-through motion. This bi-stable behavior is desirable in various applications such as micro-actuators [4,5,6,7], micro-resonators [13], bifurcation-based gas sensors [14], micro-tweezers [15], micro-valves [16], micromechanical based memories with curved electrode arrangement for reduced actuating voltage [17], micro-switches [18], and large stroke and buckling based smart applications [19].

**Figure 1:**
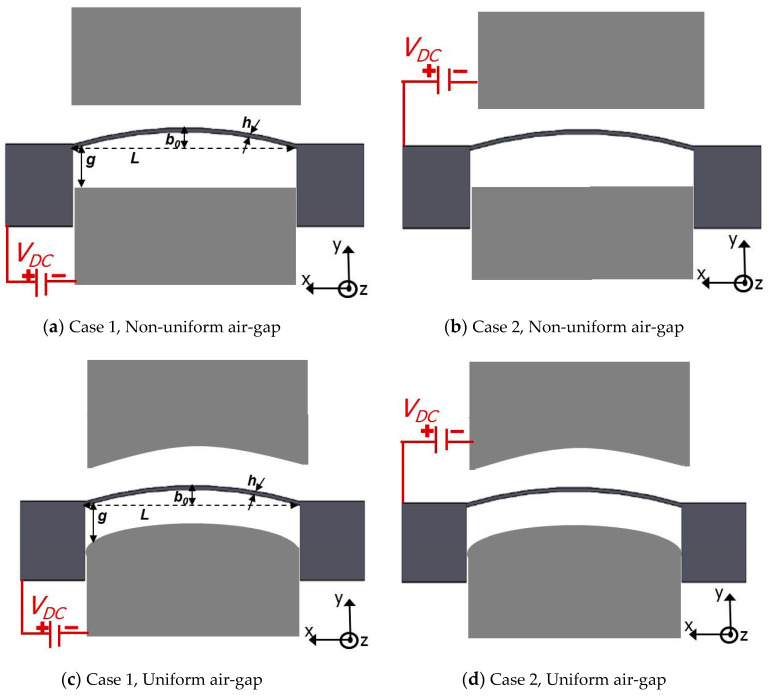
Schematics of electrostatically actuated in-plane COSINE-shaped resonators. (**a**) Case 1 and (**b**) Case 2: the micro-beams and the stationary electrodes are separated with non-uniform air-gap (*g*). (**c**) Case 1 and (**d**) Case 2: the micro-beams and the electrodes are separated with a uniform *g*.

In addition, numerous research works [20,21,22,23,24,25] examined the effect of the curved micro-beam geometry (length, thickness, etc…) as well as its initial shape (ARC, COSINE, V, etc…) on the snap-through and pull-in instabilities. Indeed, the static and dynamic behaviors of initially-curved micro-beam-based resonators actuated electrically with non-uniform gaps, Figure 1a,b, have been extensively examined [26,27,28,29] demonstrating improved tunability as compared to straight micro-beams with uniform gaps. Krylov et al. [29] conducted theoretical and experimental investigations of COSINE-shaped microbeams actuated by DC loads and have examined the bistability condition for these electrostatically actuated shallow arched micro-beams. Our group have previously investigated other curved micro-beam shapes such as the ARC and V-shaped designs [27] and through examining their static and dynamics behavior, these designs were shown to be useful in numerous applications ranging from switches to pressure and magnetic sensors [30,31]. In the literature the dynamic behavior of shallow arched COSINE-shaped microbeams have been extensively studied assuming non-uniform airgap profiles. In [32], we have investigated the V-shaped micro-beam with uniform airgap.

On the other hand, electrically actuated COSINE-shaped actuators with uniform gap-size, in which the stationary electrodes are curved like the arch, Figure 1c,d, remain relatively un-explored despite few attempts such as [33]. Consequently, this investigation aims to examine the static and dynamic behaviors, the pull-in instability, and snap-through of in-plane electrostatic COSINE-shaped arch and compare its behaviors with the non-uniform gap designs. We examine an alternative configuration to the traditional one of placing the flexible bi-stable micro-beam in front of the concave (forward) side of a beam rather than the convex (backward) side. In addition, uniform gap-size design is to be investigated for more flexibility to switch between snap-through and pull-in instabilities and with the hope of reducing the switching voltages as compared to the traditional non-uniform air-gap actuation design. 

## 2. Problem Formulation

**Figure 2:**
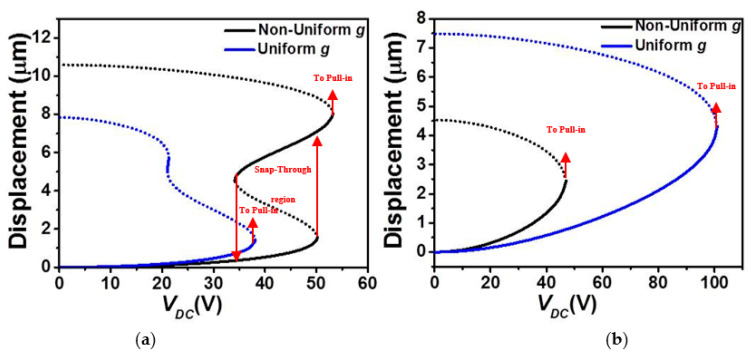
The static displacement of micro-beams with a uniform and a non-uniform *g* under DC electrostatic actuation (*V_DC_*) for (**a**) Case 1 and (**b**) Case 2 (solid: stable, dashed: unstable).

As shown in Figure 1, the micro-resonator design consists of an in-plane COSINE-shaped micro-beam clamped from both sides. A DC voltage *V_DC_* is assumed to be applied between the micro-beam and the stationary electrode resulting into an electrostatic attractive force, which pushes the flexible micro-beam towards the stationary one. Figure 1 portrays four stationary electrodes actuation arrangements offering four different ways of designs and informative comparisons. The first design with flexible and stationary electrodes are separated with non-uniform air-gap (Case 1: lower actuation, Figure 1a, and Case 2: upper actuation, Figure 2b), and the second design has both flexible curved micro-beam and its actuating electrodes separated with a uniform air-gap (Case 1: lower actuation, Figure 2a, and Case 2: upper actuation, Figure 2b). It is worth noting here that for Case 2 in both arrangements, as the DC load increases the movable electrode stroke increases until reaching the pull-in instability. In contrary, in the Case 1, the COSINE-shaped micro-beam might undergo both the snap-through and pull-in instabilities together or directly undergoes the pull-in instability.

Next, the nonlinear equations of the COSINE-shaped micro-beam, Figure 1, under the effect of the actuating forces are presented. The micro-beam is assumed to be made from Silicon with ρ as its material mass density and E its effective Young’s modulus of elasticity. The rectangular cross-sectional based micro-beam is of the following geometrical properties: *L* (Length), *b* (width), and *h* (thickness), and with *A* = *bh*, and *I* = *bh*^3^/12 are, respectively, its cross-sectional area and second moment of inertia. Several equations can be assumed to designate the initial curved profile of the investigated micro-beam [29]. Here, we assume the below COSINE-shaped expression:

(1) $$w_{0}\left(x\right)=b_{0}\left[1-\cos\left(\frac{2\pi x}{L}\right)\right];\;\mathrm{for}\;0\leq x\leq L.$$

In the above, $b_{0}$ designates the initial rise at the mid-point of the actual micro-beam initial profile.

Assuming a nonlinear model based on the Euler–Bernoulli beam theory, the in-plane deflection in the y-direction w of the COSINE-shaped micro-beam and its boundary conditions are governed by [29]:

(2) $$\left\{ \begin{array}{c} EI\frac{\partial^{4}w\left(x,t\right)}{\partial x^{4}}+\rho A\frac{\partial^{2}w\left(x,t\right)}{\partial t^{2}}+\tilde{c}\frac{\partial w\left(x,t\right)}{\partial t}=\frac{EA}{2L}\left(\int_{0}^{L}\left[{w^{\prime }}^{2}-2w^{\prime }{w^{\prime }}_{0}\right]dx\right)\left(w^{\prime \prime }-{w^{\prime \prime }}_{0}\right)+F_{e}\left(w\left(x,t\right)\right)\\ {w\left(x=0\right)|}_{\forall t}{=w\left(x=L\right)|}_{\forall t}=0;{\frac{\partial w\left(x=0\right)}{\partial x}|}_{\forall t}{=\frac{\partial w\left(x=L\right)}{\partial x}|}_{\forall t}=0;\;\;\;\;\;\;\;\;\;\;\;\;\;\;\;\;\;\;\;\;\;\;\;\;\; \end{array}\right.;$$

where *t* is time, *x* is space, and $\tilde{c}$ represents the overall viscous damping coefficient. In Equation (2), the symbols ${\left(.\right)}^{\prime }$ and ${\left(.\right)}^{\prime \prime }$ denote the first and second rate of change, respectively, with respect to *x*. The right-hand side of Equation (2) represents the resultant nonlinear force to model both the mid-plane stretching and initial curvature effects. The function $F_{e}$ represents the resultant electric load with neglecting the fringing-fields effect, which was examined and found to be of negligible outcome in [34,35]. Assuming a parallel-plates theory, the resultant electrostatic force, which has mainly a space-dependent profile, can be written as follows [34,35]:

(3) $$F_{e}\left(w\left(x,t\right)\right)=\frac{\pm \varepsilon_{0}bV_{DC}^{2}}{2{\left(g\left(x\right)\pm w\left(x,t\right)\right)}^{2}};$$

where $\varepsilon_{0}$ is the permittivity of the air and g(x) denotes the initial uniform gap size profile between the micro-beam and the electrodes g(x)=g for the cases of Figure 1c,d and equal to $g\left(x\right)=g\pm w_{0}\left(x\right)$ for the cases of Figure 1a,b. The ± signs in the numerator and denominator are to differentiate the lower and upper actuation cases, receptively. For example, Case 1 assumes “+” sign and Case 2 undertakes “−” sign in both numerator and denominator, respectively.

Equation (3) is next substituted into Equation (2) and the resultant equation is discretized using the Galerkin technique [34]. For this, the micro-beam deflection is expanded as follows:

(4) $$w\left(x,t\right)=\sum_{k=1}^{M}\psi_{k}\left(x\right)q_{k}\left(t\right);.$$

In the above Equation (4), the functions $\psi_{k}$ represent the mode-shapes of an un-forced and un-damped doubly-clamped straight beam and $q_{k}$ are their respective time-dependent modal coordinate amplitude. This forms a reduced-order model consisting of *M*-differential equations, which are numerically solved [34].

The static deflection is first calculated numerically by setting all time dependent terms in the ROM differential equations equal to zero [34]. Then the modal amplitudes $q_{k}$ are replaced by unknown constant quantities *a_i_*. This results in a system of nonlinear algebraic equations in terms of those coefficients, which is then solved numerically using the Newton-Raphson method as it is simple to be implemented and relatively less computationally expensive. Each equilibrium point can be stable or unstable and this query of stability is very important for any dynamical systems [2,36]. The entire static curve is computed by conducting a sweep through its constitutive equilibrium points [36] but before proceeding further, it should be noted that the stability analysis in the below would be a local one because the original nonlinear system will be linearized to compute the eigenvalue problem [2]. Formerly, the eigenvalue problem is solved to get the proper microbeam natural frequencies and this through numerically computing the eigenvalues of the Jacobian matrix of the generated ROM. For this, a Jacobian matrix-based eigenvalue problem is first constructed from the ROM equations to compute the in-plane flexure natural frequencies of the micro-beam at a given DC voltage after substitution of the equilibrium solutions into the Jacobian matrix [2].

## 3. Results and Discussion

As a case study, we consider the COSINE-shaped micro-beam with the material and geometrical properties summarized in Table 1. A convergence examination was already carried out in [26] and five modes was found to be satisfactory for numerical convergence (*M* = 5).

## 4. Static Analysis

Figure 2 depicts the effect of the stationary electrode arrangement on the absolute maximum static deflection of the COSINE-shaped micro-beam of Table 1 as varying the DC load. Figure 2a illustrates that for the case of non-uniform gap-size and lower actuation (Case 1, Figure 1a), the micro-beam undergoes a continuous snap-through first and then pull-in. Nevertheless, for the case of uniform gap-size and lower actuation (Case 1, Figure 1c), the same micro-beam snaps-through and pulls-in right away while increasing the DC voltage. The figure also shows that the uniform gap-size case is registering lower threshold voltage to snap (approx. 38.1 *Volt*) as compared to the non-uniform gap-size case (approx. 50.2 *Volt*) which is approximately a drop of 25%. Such trend was previously reported in [33]. It is worth noting that, for this lower actuation design, there is a band of DC loads, where two stable micro-beam’s displacements are co-existing: one original stable solution (concaved up shape) and a buckled state (concaved down shape). Comparing closely the simulated cases in Figure 2a, the static behavior changed from a bi-stable one for the non-uniform case, where the pull-in voltage is greater than the snap-through voltage, to a dynamically bistable for the case of the uniform air-gap size, where the pull-in threshold voltage is lower than the snap-through voltage. This was reported previously in [37].

Comparing now the cases of upper electrode arrangement, increasing further the actuating electrostatic force, the absolute maximum static displacement increases until reaching the pull-in instability for both uniform and non-uniform gap-size outlines, Figure 2b. In both cases, the electrostatic force further changes (increases) the initial curvature of the micro-beam thus stiffening it further and the micro-beam undergoes an immediate pull-in without any snap-through behavior. From the same figure, one can also note an increase in the pull-in voltage threshold moving from the non-uniform electrode case (approx. 47 *Volt*) to the uniform electrode cases (approx. 101 *Volt*) which is approximately a 53% drop.

**Figure 3:**
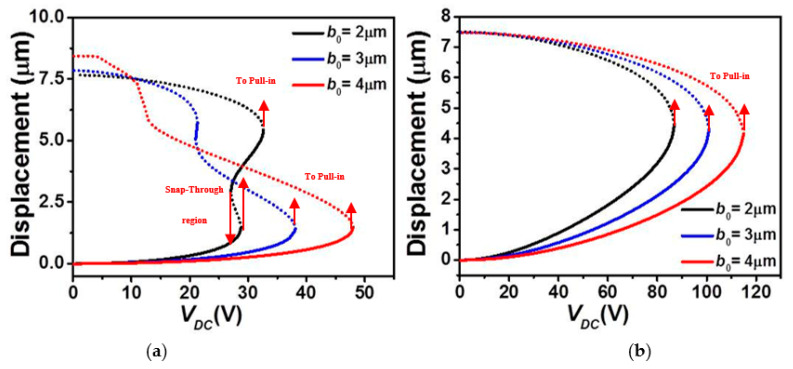
The static displacement of the micro-beam of Table 1 with a uniform *g* under *V_DC_* for various values of initial rise (*b*_0_) for (**a**) Case 1 and (**b**) Case 2 (solid: stable, dashed: unstable).

**Figure 4:**
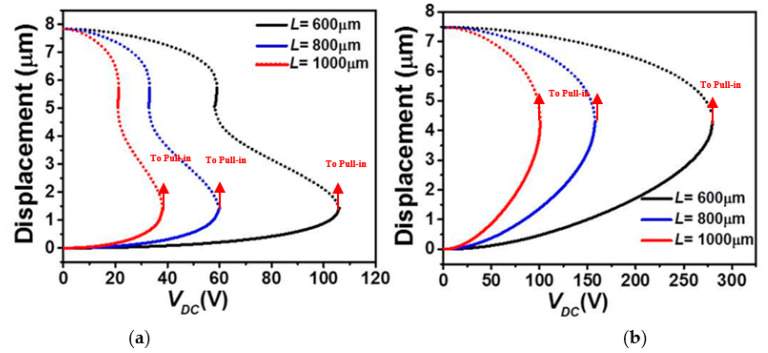
The static displacement with a uniform *g* and for various values of length (*L*) under *V_DC_* for (**a**) Case 1 and (**b**) Case 2 (solid: stable, dashed: unstable).

In Figure 3 and Figure 4, we examine the effect of the micro-beam’s mid-point initial rise and length, respectively, on its static behavior considering the lower and upper stationary electrode’s arrangements with uniform air-gap. It is worth mentioning that when increasing the elevation, the higher order modes, including asymmetric modes, will get involved in the overall structural behavior of the micro-beam resulting into possible symmetry breaking [38,39]. Since the goal in the below analysis is to study the effect on the symmetric regime, we will inhibit the effect of the asymmetric modes. This can always be done through assuming a double beam structure of identical elevation to prevent any contribution from the asymmetric modes [40]. Figure 3a and Figure 4a demonstrate that, for the case of lower actuation, the snap-through threshold voltage grows with the increase in the mid-point’s initial rise value and decrease of the effective length of the micro-beam. In both scenarios, it is clear that the overall effective stiffness of the micro-beam increases before snap-through and then reduces when reaching the pull-in state. The same trend, but with an immediate pull-in behavior, is found in the cases of the upper actuation designs, Figure 3b and Figure 4b. Indeed, from both figures, one can see a noticeable increase in the pull-in threshold voltage with the growth of the mid-point’s initial rise value and decrease of the effective length of the micro-beam of Table 1.

## 5. Eigenvalue Problem Analysis

**Figure 5:**
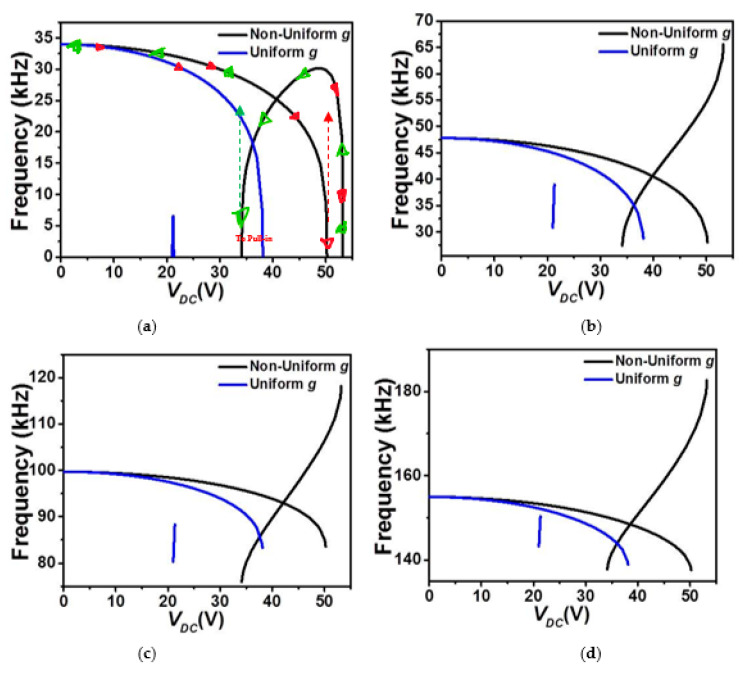
The variation of the (**a**) first, (**b**) second, (**c**) third, and (**d**) fourth in-plane flexure natural frequencies for Case 1 as varying *V_DC_* and for uniform and non-uniform air-gaps (*g*).

**Figure 6:**
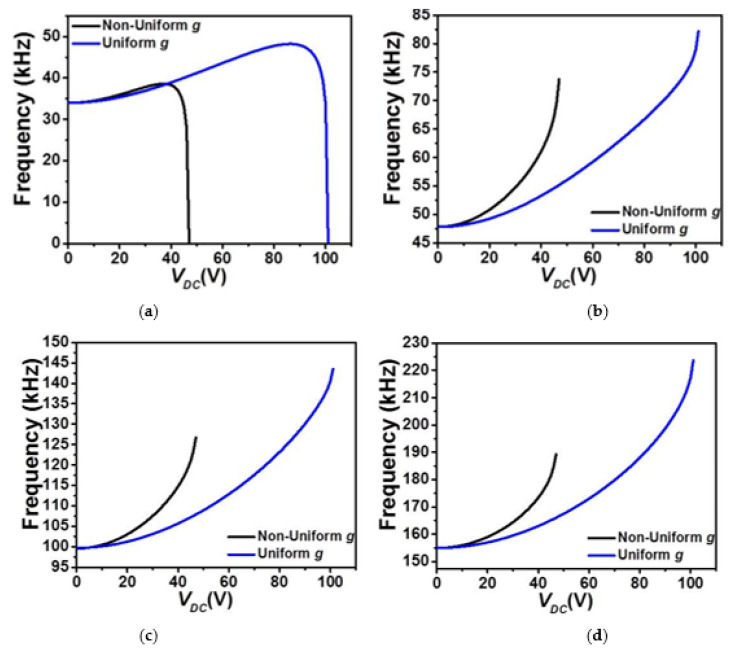
The variation of the (**a**) first, (**b**) second, (**c**) third, and (**d**) fourth in-plane flexure natural frequencies for Case 2 as varying *V_DC_* and for uniform and non-uniform *g*.

In order to further investigate the various designs, we examine next the variation of the eigenvalues (in-plane flexure natural frequencies) of the micro-beam with the DC load. Figure 5 and Figure 6 show the effect of the DC load and both gap-size configurations (uniform and non-uniform cases) on the variation of the four lowest natural frequencies of the COSINE-shaped micro-beam for both cases of upper and lower electrostatic actuation, respectively. In both cases of uniform and non-uniform air-gap size, the first four in-plane flexure natural frequencies are all displaying a constant decrease until reaching the snap-through instability where the fundamental (lowest) frequency, Figure 5a, drops instantaneously to zero. For this configuration, this behavior proves the dominance of the quadratic nonlinearity, tending to soften the micro-structure, arising from both the initial curvature and the actuating force. For the case of the non-uniform air-gap size, since the micro-beam still has enough room to snap and then reaches its second snap state, its fundamental frequency starts to increase after snap-through as the cubic nonlinearity arising from the mid-plane stretching is activated accordingly and then goes back to zero when reaching the pull-in state. This is not the case of the uniform air-gap size, where the micro-beam goes to the pull-in right after the snap-through.

In contrast, for the cases of upper actuation electrodes patterns where the micro-beam is closer to the actuating electrodes, all four lowest frequencies are showing an increase, Figure 6, until reaching the pull-in instability where only the lowest (fundamental) in-plane flexure natural frequency drops to zero accordingly, Figure 6a, for both uniform and non-uniform air-gap size cases. The increase of the natural frequency is a clear mark that the linear part of the micro-beam cubic nonlinearity (mid-plane stretching effect) dominates the other types of nonlinearities in these examined cases until reaching the pull-in instability where the electrostatic force takes the lead pushing its fundamental frequency to zero around the pull-in state. These results demonstrate potentials of using COSINE-shaped micro-beams with uniform air-gap sizes to achieve low snap-through threshold voltage while keeping a relatively high tunability. These designs could be very promising in numerous microsystem’ applications requiring large stroke and buckling based smart devices such as micro-actuators, switches and resonators, bifurcation-based gas sensors, micromechanical based memories with curved electrode arrangement for reduced actuating voltage.

**Figure 7:**
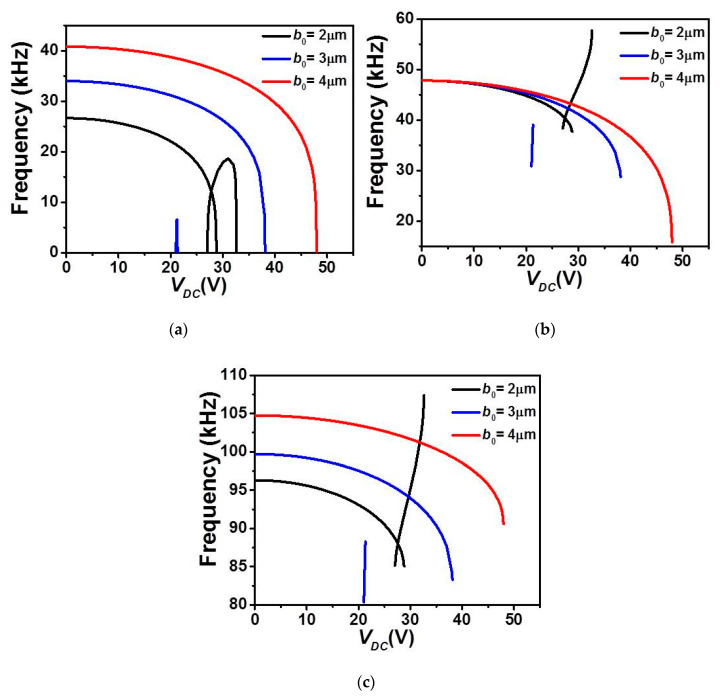
The variation of the (**a**) first, (**b**) second, and (**c**) third in-plane flexure natural frequencies for Case 1 as varying *V_DC_* with uniform *g* and for various values of *b*_0_.

**Figure 8:**
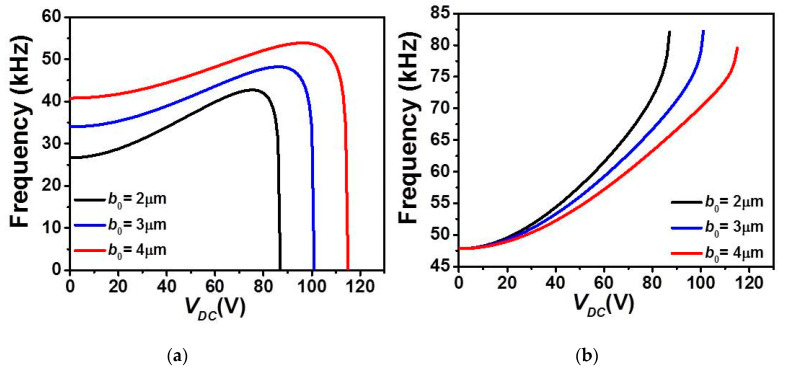

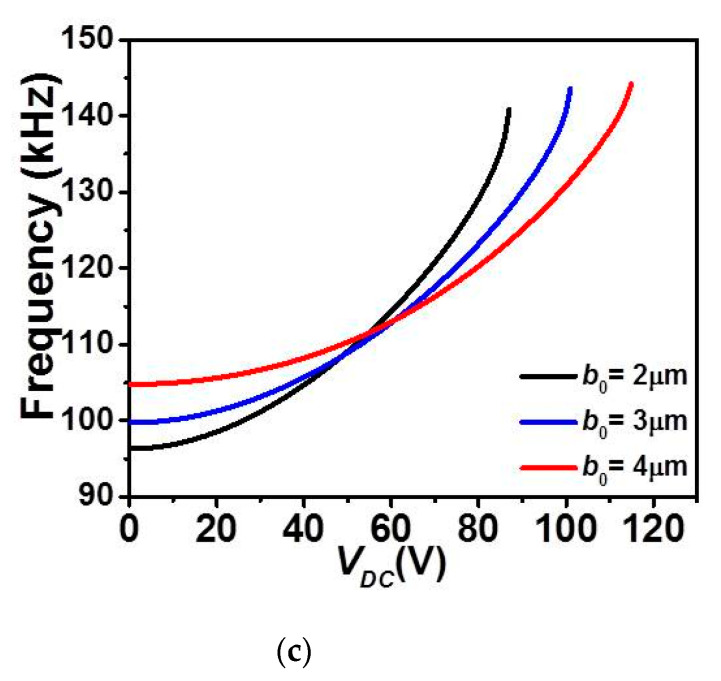
The variation of the (**a**) first, (**b**) second, and (**c**) third in-plane flexure natural frequencies for Case 2 as varying *V_DC_* with uniform *g* and for various values of *b*_0_.

Next, the variation of the first three in-plane flexure natural frequencies for the micro-beam of Table 1 with DC load assuming a uniform air-gap size and while varying its mid-point initial rise *b*_0_, are displayed in Figure 7 and Figure 8 for the upper and lower actuation arrangements, respectively. Figure 7 shows that for all cases of *b*_0_, the natural frequencies decrease and the fundamental one drops to zero when snap-through and/or pull-in instability occurs, Figure 7a–c. For higher *b*_0_ (3 and 4 µm cases), a decreasing trend is noted for the three frequencies and with only a unique drop in the fundamental when the micro-beam reaches the snap-through instability. For the case of *b*_0_ = 2 µm, the micro-beam undergoes both snap-through and pull-in instability with two drops to zero in its lowest fundamental frequency, and with a decreasing trend before snap-through and increasing trend after the snap-through.

On the other hand, Figure 8 clearly indicates a nonlinear increase in all first three in-plane flexure natural frequencies with the DC load for the case of upper actuation arrangement and for all *b*_0_ cases. The fundamental frequency, Figure 8a shows an increase for a wider range DC voltage and then drops to zero only near the pull-in state. In addition, as *b*_0_ is increased, the increase of the fundamental frequency is showing a little decline, therefore less frequency tunability in the three lowest natural frequencies, Figure 8a–c.

**Figure 9:**
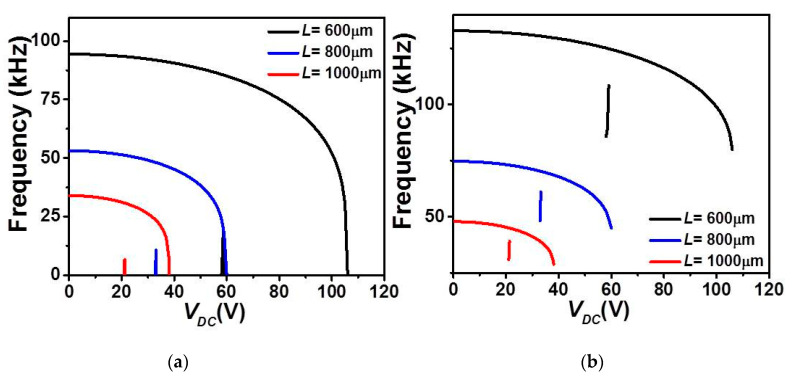

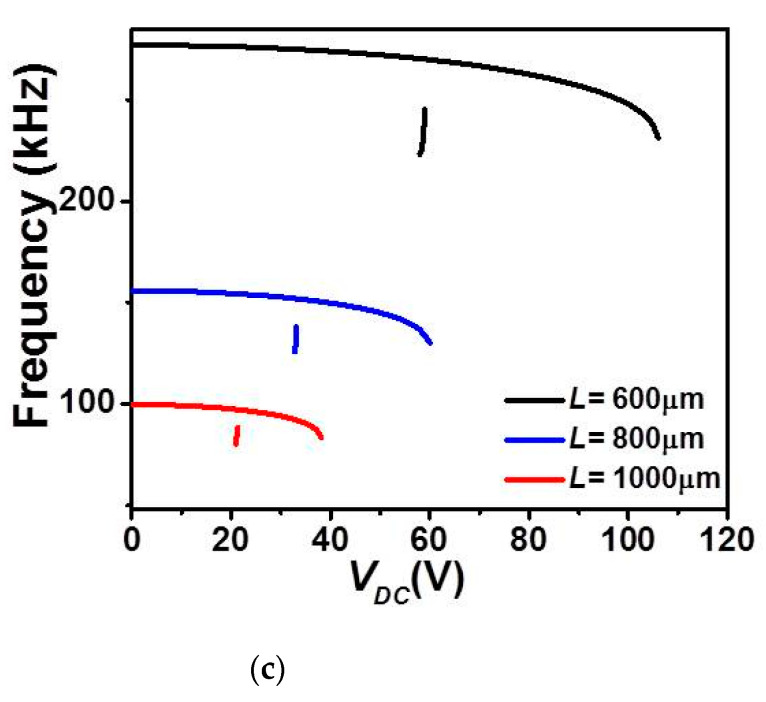
The variation of the (**a**) first, (**b**) second, and (**c**) third in-plane flexure natural frequencies for Case 1 as varying *V_DC_* with uniform *g* and for various values of *L*.

**Figure 10:**
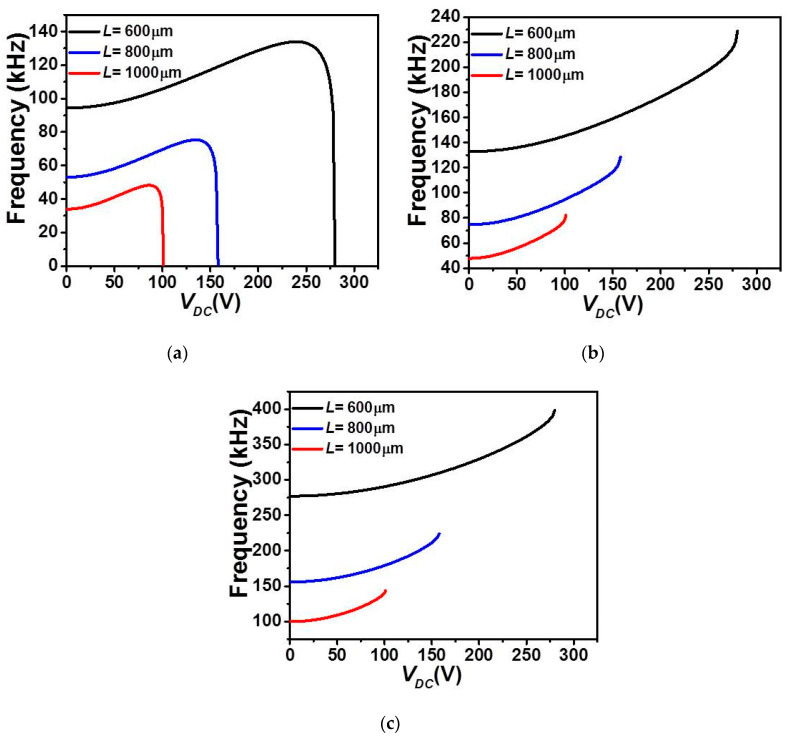
The variation of the (**a**) first, (**b**) second, and (**c**) third in-plane flexure natural frequencies for Case 2 as varying *V_DC_* with uniform *g* and for various values of *L*.

Finally, Figure 9 and Figure 10 illustrate the effect of length *L* on the first three in-plane flexure natural frequencies of the micro-beam of Table 1. It can be seen that for the case of lower actuation arrangement, Figure 9, all three natural frequencies decreased and only the fundamental one drops to zero when snap-through occurs. Beyond snap-through, and for all cases, all frequencies are not anymore present as pull-in state occurs right after the snap-through instability. On the other hand, it is shown in Figure 10 that all three frequencies are nonlinearly increasing with the DC load for the case of upper actuation arrangement and for all different assumed lengths. Indeed, the fundamental frequency, Figure 10a shows a growth for several DC load amplitudes and then drops to zero only near the pull-in instability. In addition, as *L* is decrease, the increase of the fundamental frequency is showing a non-negligible growth, therefore higher frequency tunability in the three lowest natural frequencies, Figure 10a–c. Based on these discussed results, one can adjust the geometry of the micro-structure to meet specific applications requiring adjustable tunability and larger strokes before reaching any electro-structural instability such as bifurcation-based micro-sensors and micro energy harvesters.

## 6. Conclusions

In this research work, the static and eigenvalue problem behaviors of in-plane clamped-clamped COSINE-shaped micro-beams under DC load assuming different electrostatic actuation arrangements ranging from uniform and non-uniform air-gap size profiles were examined. The simulated results show that different static behavior and diverse fundamental frequencies tunability can be obtained when moving from one actuation arrangement to another. We have also explored the major effects of few geometric parameters (beam mid-point initial rise and its effective length) on the snap-through and pull-in instabilities occurrences. The simulations showed that the initial rise and the length of micro-beam have significant effects on the structural (mainly the snap-through and pull-in voltages) and tunability behavior of COSINE-shaped micro-beam assuming a uniform air-gap size configuration. The discussed results showed promising potential to use such designs in micro-switching application (requiring low actuation voltages) and micro-actuation application requiring higher stroke with instability occurring at higher DC voltage values thus validating the advantage of curved electrodes in decreasing the fundamental threshold voltages (pull-in and snap-through) by certain percentages. In addition, the simulated results in this work demonstrated that the bi-stability snap-through behavior in these designs may or may not happen whereas the pull-in instability always exists. Indeed, to undergo the bi-stable like snap-through behavior, the COSINE-shaped uniform air-gap size design micro-beam would have a relatively lower initial rise with large effective length distance. For this, one can pick the most optimized geometry of the micro-structure to meet certain specific applications where some requiring high tunability, and other higher stroke with low actuation amplitudes.

## Figures and Tables

**Table 1:** Cosine-shaped micro-beam (material and geometrical properties of doped single-Crystal Silicon) for types 1 and 2 of Figure 1.

Material Properties	Value	Geometrical Properties	Value
Mass density ρ (kg/m^3^)	2332	Thickness *h* (µm)	2
Effective Young’s modulus E (GPa)	154	Width *b* (µm)	25
Poisson’s ratio	0.28	Length *L* (µm)	800
		Initial rise *b*_0_ (µm)	3
		Vertical air-gap *g* (µm)	8

## Data Availability

Data are contained within the article.
